# Construction and Characterization of a New Recombinant Vector to Remove Sulfate Repression of *dsz* Promoter Transcription in Biodesulfurization of Dibenzothiophene

**DOI:** 10.3389/fmicb.2018.01578

**Published:** 2018-07-17

**Authors:** Somayeh Khosravinia, Mahmood A. Mahdavi, Reza Gheshlaghi, Hesam Dehghani, Behnam Rasekh

**Affiliations:** ^1^Department of Chemical Engineering, Ferdowsi University of Mashhad, Mashhad, Iran; ^2^Stem Cells and Regenerative Medicine Research Group, Institute of Biotechnology, Ferdowsi University of Mashhad, Mashhad, Iran; ^3^Division of Biotechnology, Faculty of Veterinary Medicine, Ferdowsi University of Mashhad, Mashhad, Iran; ^4^Microbiology and Biotechnology Research Group, Research Institute of Petroleum Industry, Tehran, Iran

**Keywords:** biodesulfurization, biocatalyst, transcription, *dsz* promoter, sulfate repression, recombinant vectorpCom8

## Abstract

Biodesulfurization (BDS) is an environmentally friendly desulfurizing process with the potential of replacing or adding to the current expensive technologies for sulfur removal from fossil fuels. The BDS, however, still suffers from low biocatalyst activity. One reason is repression of *dsz* promoter transcription in presence of inorganic sulfate that impedes translation of Dsz enzymes required for desulfurization pathway. One approach to solve this problem is replacing the native promoter with a new promoter that is no longer repressed. In this study, *dsz* genes from desulfurizing strain *Rhodococcus* sp. FUM94 was cloned in an alkane responsive promoter, pCom8, and expressed in *Escherichia coli* BL21 (DE3) as a host. The recombinant was not susceptible to inorganic sulfate in the culture medium. Desulfurizing activity of recombinant strain versus wild type indicated that in a sulfate containing medium, BDS yield of recombinant increased from 16.0% ± 0.9 to 34.0% ± 1.9% when dibenzothiophene (DBT) concentration (dissolved in ethanol) increased from 25 to 100 ppm. Also, 2-hydroxy biphenyl (2-HBP) production rate improved 8.5-fold (from 0.302 ± 0.020 to 2.57 ± 0.14 mmol 2-HBP (kg DCW)^-1^ h^-1^) at the same DBT concentration range. This is while no 2-HBP production was detected in FUM94 biphasic reaction. In a sulfate-free medium, wild type strain demonstrated desulfurization activity, but decreasing with the increase of DBT concentration dissolved in *n*-tetradecane. Whereas, the recombinant strain demonstrated increasing desulfurizing activity in a sulfate-containing high DBT concentration environment. Overall, the result of this molecular manipulation can be considered as a step forward toward commercialization of BDS technology.

## Introduction

Emission of certain hazardous pollutants such as sulfur oxides into the atmosphere resulting from the combustion of petroleum causes environmental impacts such as air pollution, and human health problems (Dube et al., 2014). The selective removal of sulfur from petroleum can reduce these impacts. However, some aromatic sulfur compounds such as dibenzothiophene (DBT) and its alkylated derivatives are resistant to conventional hydrodesulfurization (HDS) technology. Biodesulfurization (BDS) is the enzymatic cleavage of carbon–sulfur bonds in compounds recalcitrant to HDS. Despite the economic benefits of BDS over rival technologies for sulfur removal, this technology suffers from a major obstacle, i.e., low biocatalyst activities that prevents it from being used as a commercial technology (Kilbane, 2006).

In the past decades, considerable efforts have been made to understand and overcome the limitations to the desulfurization activity of the biocatalysts. Basically, desulfurizing bacteria remove sulfur from refractory compounds (DBT and alkylated DBTs) in the sulfur specific 4S pathway encoding by *dsz*ABC operon. One reason for the limited activity is that Dsz enzymes are sulfate-starvation-induced proteins (Tanaka et al., 2002) and consequently desulfurization activity is repressed by preferred sulfur source, including inorganic sulfate and the sulfur containing amino acids methionine and cysteine (Piddington et al., 1995; Matsui et al., 2002). These sulfur-containing compounds do not affect the activity of desulfurizing enzymes, rather; they strongly repress transcription from *dsz* promoter (Li et al., 1996). On the other hand, since the concentration of Dsz enzymes could be considered as the rate limiting factor, many efforts were accomplished to achieve very high levels of expression of the desulfurizing genes by supplying multiple copies of *dsz* genes and or using of alternative strong promoters (Ma et al., 2006). Therefore, genetic engineering tools have been used to construct an efficient recombinant biocatalyst through the replacement of the native *dsz* promoter alleviated sulfur repression. Thereby, several studies employed *dsz* promoter replacement approach to improve desulfurization yield. Gallardo et al. (1997) subcloned *dsz* cluster into the broad-host-range plasmid pVLT31 under the control of Ptac promoter using a soil strain, *Pseudomonas aeruginosa* PG201 as an expression host. Matsui et al. (2002) constructed a recombinant strain by using the putative *rrn* promoter region from *Rhodococcus* sp. strain T09 and expressed the Dsz phenotype in this strain. Serbolisca et al. (1999) replaced the native *dsz* promoter with the control region of the neomycin phosphotransferase gene and then introduced the construct into the *Arthrobacter* sp. DS7. Ma et al. (2006) expressed *dsz* genes from *Rhodococcus erythropolis* DS-3 efficiently under the spac promoter in *Bacillus subtilis* as an industrially optimal host. Desulfurization activity of *B. subtilis* recombinant was 16.2 mg DBT l^-1^ h^-1^ that was more than that of wild type DS-3 (13.1 mg DBT l^-1^ h^-1^). Shavandi et al. (2009) utilized the *lac* promoter for self-cloning of *dsz* genes in *Gordonia alkanivorans* RIPI90A. It resulted in 2.67-fold desulfurization activity of recombinant resting cells in comparison to native strain. All reported desulfurization improvements in the above-mentioned investigations were attributed to replacement of native promoter with a known promoter from other sources.

In addition, organic solvent inducible promoter PalkB in the alkane oxidation system of *Pseudomonas oleovorans* was used to construct an alkane responsive expression vector pCom8 bearing AlkS gene. PalkB promoter is regulated by the transcriptional activator AlkS and can be induced by the C7-C12 n-alkanes, alkenes, ethyl acetate, and dicyclopropylketone (DCPK). The vector pCom8 yields high induction and expression levels of both cytoplasm and membrane proteins (Smits et al., 2001). It is very advantageous, because these organic solvents are inexpensive and available in biphasic reaction of BDS, while isopropyl-β-D-1-thiogalactopyranoside, the inducer of most of expression vectors and some induction methods such as heat shock activation are expensive and cumbersome on a large scale. Therefore, vector pCom8 containing PalkB promoter has the potential to be used as a replacing promoter for the expression of *dsz* genes in cases where sulfate repression is problematic. This is specifically important as the promoter is induced by organic solvents that are abundant in hydrocarbon fuels such as diesel and gasoline.

In this study, desulfurizing gene cluster *dsz* of *Rhodococcus* sp. FUM94 was cloned in vector pCom8 to construct recombinant vector pCom8ABC. The new construct was introduced into *E. coli* BL21 (DE3) as a host strain. The expression of *dsz* genes in the new host under the control of PalkB promoter was confirmed through desulfurization activity of recombinant strain in presence of sulfate.

## Materials and Methods

### Bacterial Strains and Cultivation Conditions

The genes responsible for DBT degradation (*dsz* operon) were obtained from *Rhodococcus* sp. FUM94, a DBT-desulfurizing bacterium that uses the 4S pathway and designated and characterized in our previous study (Khosravinia et al., 2018). *Escherichia coli* DH5α was used for general cloning and *E. coli* BL21 (DE3) was used as heterologous host strain for the expression *dsz* genes from plasmid pCom8 (7639 bp), an *E. coli*–*Pseudomonas* shuttle vector that confers gentamicin resistance. **Table 1** provides a listing of plasmids and strains used in this study.

**Table 1:** The listing of plasmids and strains used in this work.

Resource	Characteristics
**Strains**	
*Rhodococcus* sp. FUM94	Wild type desulfurizing strain
*E. coli* DH5α	Cloning host strain
*E. coli* BL21 (DE3)	Expression host strain
**Plasmids**	
pCom8	*E. coli–Pseudomonas* shuttle expression vector with *PalkB*, Gm^r^, *oriT*, *alkS*
pCom8ABC	pCom8 with *dsz* gene cluster in *Eco*RI–*Hin*dIII sites


For selection of *E. coli* transformants, gentamicin was added to LB agar (1.5%, m/v) plates to final concentrations of 10 μg mL^-1^. The basal salt medium (BSM) supplemented with metal traces was used for cultivation of strain FUM94 and *E. coli* BL21 transformants in DBT desulfurization assays. BSM composition was as follows (Izumi et al., 1994):4 g glycerol, 0.5 g KH_2_PO_4_, 4 g K_2_HPO_4_, 1 g NH_4_Cl, 0.2 g MgCl_2_. 6 H_2_O, 0.02 g CaCl_2_, 0.01 g NaCl and 10 mL metal solution in 1000 mL of deionized water. Metal solution contained (per liter) 0.5 g FeCl_2_. 4H_2_O, 0.5 g MnCl_2_. 4H_2_O and 0.05 g ZnCl_2_ and 120 mmol HCl. The pH was adjusted to 7.2 prior to autoclaving. In order to prevent metal complex formation, the medium and metal solutions were autoclaved separately and then metal solution was added to the medium. Gentamicin (10 μg mL^-1^) and yeast extract (0.1%) were added to BSM for the *E. coli* cultures.

### Chemicals

Dibenzothiophene (98%), 2-hydroxy biphenyl (2-HBP), *n*-tetradecane, and acetonitrile were purchased from Merck Chemicals (Germany) and, DCPK (96%) from Alfa Aesar (United States). Acetonitrile was of liquid chromatography grade and other chemicals were of analytical grade. DNA ladders, T4 DNA ligase and restriction endonucleases were obtained from Fermentas Ltd. and Protein ladder was purchased from Eurx Ltd.

### PCR Amplification and Cloning of *dsz* Genes

Restriction digestion, agarose gel electrophoresis, isolation of plasmids, and other DNA manipulations were carried out according to standard protocols (Sambrook and Russell, 2001) or by following the manufacturer protocols. The *dsz* gene cluster from genomic DNA of *Rhodococcus* sp. FUM94 was amplified with a PCR primer pair that was designed based on known sequence of *dszABC* of *R. erythropolis* IGTS8 (GenBank accession number U08850). The sequences of primers were:

dszF: 5′-GGAGAATTCAAGGACGCATACGCGATGAC-3′ and
dszR: 5′-GGAAAGCTTTCAGATCCTCAGGAGGTGAAG-3′.

The *Eco*RI and *Hin*dIII restriction sites in forward and reverse primers underlined, respectively.

The amplified product contained 15 bases upstream of dszA and eight bases downstream of dszC. The *Eco*RI site of the dszF primer is not within the putative RBS of dszA. In fact, dszF primer contained nine bases in 5′ tail that was unmatched and 20 matched bases that have overlap with five first base of dszA gene sequence.

**FIGURE 1:**
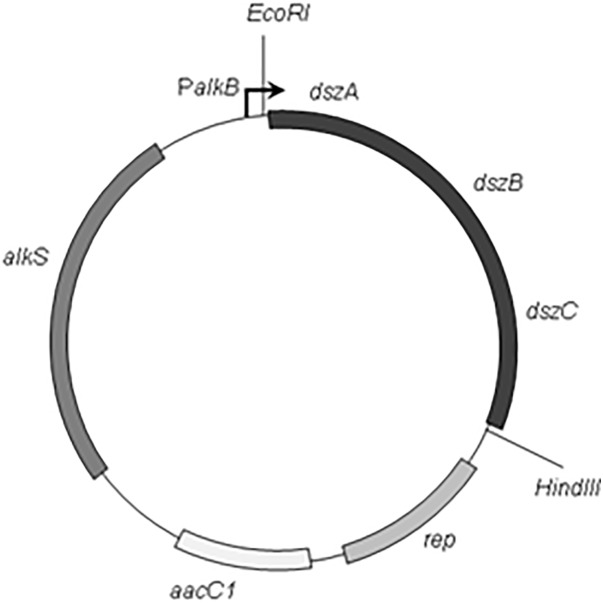
Schematic map of the plasmid pCom8ABC. Restriction sites and structural genes are indicated (The *Eco*RI and *Hin*dIII restriction sites in multiple Cloning Site of vector pCom8 were located on upstream and downstream of *dsz* cluster, dszA, dszB, and dszC encoding dibenzothiophene desulfurization enzymes from *Rhodococcus* sp. FUM94; aacC1, gentamicin resistance gene; alkS, the positive regulatory gene of promoter PalkB; rep, the *Pseudomonas* origin of replication).

The PCR amplification was set up by *Accu Power*^®^ *Pfu* PCR PreMix (Bioneer, Korea) and performed using a programmable thermal cycler (Light Cycler, Bio Rad). The reaction mixture includes 20 pmol of each primer and 100 ng of genomic DNA template in a 50 μL final volume. The thermal profile of PCR was initial denaturation at 94°C for 5 min, 30 cycles consisting of denaturation at 94°C for 30 s, primer annealing at 58°C for 30 s, and extension at 72°C for 8 min and then, a final extension for 10 min at 72°C. The 3.7 kb *dszABC* PCR product fragment was digested with *Eco*RI and *Hin*dIII. Then, the gel purified fragment was ligated into *Eco*RI–*Hin*dIII digested pCom8 vector. The resulting recombinant plasmid was designated as pCom8ABC (**Figure 1**). The construct pCom8ABC was transformed into *E. coli* DH5α competent cells by heat shock method. Then, the accuracy of cloning was verified with *Eco*RI–*Hin*dIII double digestion of construct pCom8ABC from positive colonies and subsequent agarose gel electrophoresis and also with nucleotide sequencing plasmid pCom8ABC in insert region (*dsz* cluster). After cloning verification, construct pCom8ABC was introduced into *E. coli* BL21 (DE3) competent cells to express *dsz* genes under the control of PalkB promoter in pCom8.

### Desulfurization Assays

To run the desulfurization assay, a single colony of recombinant bacterium from LB agar plate was incubated into 3 mL of LB broth in 15 mL falcon tube agitated at 150 rpm and 30°C during 12 h on a rotary shaker. Afterward, the culture was diluted into fresh LB with ratio 1:10 and agitated at the same operational conditions for another 3 h. Next, the solution was centrifuged at 4500 rpm for 10 min and the cell pellet was re-suspended in 100 mL BSM in 500 mL Erlenmeyer flask to the inoculum concentration of 0.25 g L^-1^.

For recombinant cells, in BSM medium, MgCl_2_ was replaced by 1.5 mM MgSO_4_ as the sulfur source (in the form of sulfate). To induce the expression of desulfurizing *dsz* genes, DCPK (0.05%, w/v) was added to growth medium. Growth at 30°C and 150 rpm was stopped after 24 h (at mid-logarithmic phase) and recombinant resting cells were harvested by centrifugation at 4500 rpm for 10 min. Then washed twice with 0.1 M potassium phosphate buffer (pH 7.0) and finally re-suspended in potassium phosphate buffer (pH 7.0) to reach a concentration of 2.0 g dry cells L^-1^.

The biphasic desulfurization assays were performed in falcon tubes, with total volume of 6 mL, on rotary shaker at 150 rpm and 30°C for 12 h, using a 1:3 ratio of *n*-tetradecane (organic phase) to potassium phosphate buffer (aqueous phase). *n*-Tetradecane was employed as a representative of hydrocarbons in petroleum. DBT with concentrations of 25, 50, and 100 ppm was utilized in two different ways. In a series of experiments, DBT was initially dissolved in ethanol as a co-solvent (indirectly in aqueous phase), and then added to the two-phase system. Organic phase was pure *n*-tetradecane. In another series of experiments, DBT was directly dissolved in *n*-tetradecane as organic phase. Negative control of *E. coli* BL21 carrying pCom8 plasmid without *dsz* genes insert was also grown under the same conditions.

Native desulfurizing bacterium, *Rhodococcus* sp. FUM94 was also incubated in BSM medium containing 10 ppm DBT as a sole sulfur source (30°C and 150 rpm shaking). The cells were harvested at the late logarithmic growth phase (ca. OD_660_ = 3.0) and then cell pellets were washed twice with 0.1 M potassium phosphate buffer (pH 7.0) and then re-suspended in potassium phosphate buffer (pH 7.0) to reach a concentration of 2.0 g dry cells L^-1^. This solution was introduced into biphasic desulfurization reaction, similar to BL21 strain.

In all experiments, the 2-HBP final product was quantified by high-performance liquid chromatography analysis. Desulfurization activity was evaluated based on 2-HBP specific production rate (q_p_) defined as the quantity of 2-HBP produced per hour per kilogram of dry cell weight, [mmol 2-HBP (kg DCW)^-1^ h^-1^] and BDS yield (X_BDS_) defined as the ratio of the 2-HBP produced to the initial substrate concentration.

### HPLC Analysis

Detection and quantification of 2-HBP dissolved in hydrocarbon fraction was carried out by HPLC equipped with a Kromasil C18 column (150 mm × 4.6 mm, 5 μm). A mix of Acetonitrile/water (50/50) was the mobile phase with the flow rate of 1 ml min^-1^ and detection was performed using an UV detector at 280 nm. All samples were centrifuged to separate hydrocarbon phase from the aqueous phase containing cells at 5500 rpm for 30 min at 25°C (Rashtchi et al., 2006). Then, the supernatant organic phase was injected to HPLC column. The injection volume was fitted to 50 μL. To determine the concentration of 2-HBP, the standard curves were plotted using a series of pure compound dilutions. To elute adsorbed *n*-tetradecane from the column after analysis of each sample, a washing cycle with mobile phase was run for 30 min.

### SDS–PAGE

*Escherichia coli* BL21 (DE3) cells containing pCom8ABC were grown overnight at 37°C, in a shaker at 200 rpm, in LB medium supplemented with gentamicin (10 μg mL^-1^). Bacterial cells were then diluted 1:10 in fresh LB-gentamicin medium and cultured until the absorbance reached 1.0 at 600 nm; then DCPK (0.05%, w/v) as an inducer was added to the culture medium. The cultivation temperature was shifted to 25°C to avoid the formation of insoluble protein aggregates at 37°C. Aliquots of the culture broth was taken after 5 h induction. Negative control of *E. coli* strain BL21 (DE3) carrying only pCom8 plasmid was also grown under the same conditions. Then, bacterial cells were harvested by centrifugation at 12,000 rpm for 5 min and the supernatant was discarded. The cell pellets in both groups (recombinant and negative control) were prepared to be subjected to sodium dodecyl sulfate poly acrylamide gel electrophoresis (SDS–PAGE) using standard protocols.

Briefly, the cell pellets from 5 mL culture medium were rinsed with 40 μL phosphate buffered saline pH 7.0 and mixed with 10 μL of 5× SDS–PAGE loading buffer and heated for 10 min at 95°C. Then, they were quickly centrifuged and cooled down to the room temperature and 20 μL of the supernatant (equivalent to about 20 μg total cell protein) were loaded and run on to the SDS gel containing 12.5% and 4% polyacrylamide in the separating and stacking gels, respectively. The protein bands were visualized by staining with 0.25% Coomassie brilliant blue G-250 dissolved in 50% methanol–10% acetic acid and then destained with 30% methanol–10% acetic acid.

### Statistical Analysis

The 2-HBP concentration data are average values with margin of errors at confidence level of 95% from triplicate experiments.

## Results and Discussion

In order to remove sulfate repression of the transcription from native *dsz* promoter, the *dsz* operon from *Rhodococcus* sp. FUM94 was cloned in alkane inducible vector pCom8. This vector that harbors PalkB promoter is not susceptible to sulfate presence in the culture medium and transcription from the promoter is no longer repressed in presence of sulfate. Gel electrophoresis analysis and sequencing results showed the correct insertion of *dsz* operon in vector and SDS–PAGE analysis confirmed the size of the expressed proteins. In addition, expression of *dsz* genes in the new host was verified through desulfurization activity of recombinant *E. coli* BL21 (as described below) that shows Dsz enzymes are successfully encoded and 4S pathway is fully in function.

### Construction of Expression Plasmid pCom8ABC

The *dsz* gene cluster was amplified by using PCR technique and genomic DNA of *Rhodococcus* sp. FUM94 as a template and *Pfu* polymerase. Insertion of *dsz* cluster fragment from strain FUM94 amplified by PCR in expression vector pCom8 was easily conducted in correct direction by setting appropriate restriction sites of *Eco*RI and *Hin*dIII in PCR primer pair according to multiple cloning site of vector and also applying a useful platform in the primers for restriction enzymes. It was verified by agarose gel electrophoresis results following diagnostic digestion reaction on plasmid pCom8ABC with *Eco*RI and *Hin*dIII restriction enzymes. The digestion resulted in two fragments of 3.7 kb (*dsz* cluster as the insert) and 7.6 kb (linearized pCom8 vector) that confirmed that the desired recombinant plasmid was obtained. To further confirm the construction, the whole insert (dsz genes) and its neighborhood regions (about 3.8) related to construct pCom8ABC were sequenced. Nucleotide sequence alignment of insertion region (about 3.8 kb) in pCom8ABC was performed against *dsz* genes of *Rhodococcus* sp. FUM94 (accession number of KT987928) using the web-based BLAST program of the National Center for Biotechnology Information (NCBI). It indicated that no mutation was occurred and rejected the possibility of PCR error.

### The Dsz Protein Expression and Gel Electrophoresis

**FIGURE 2:**
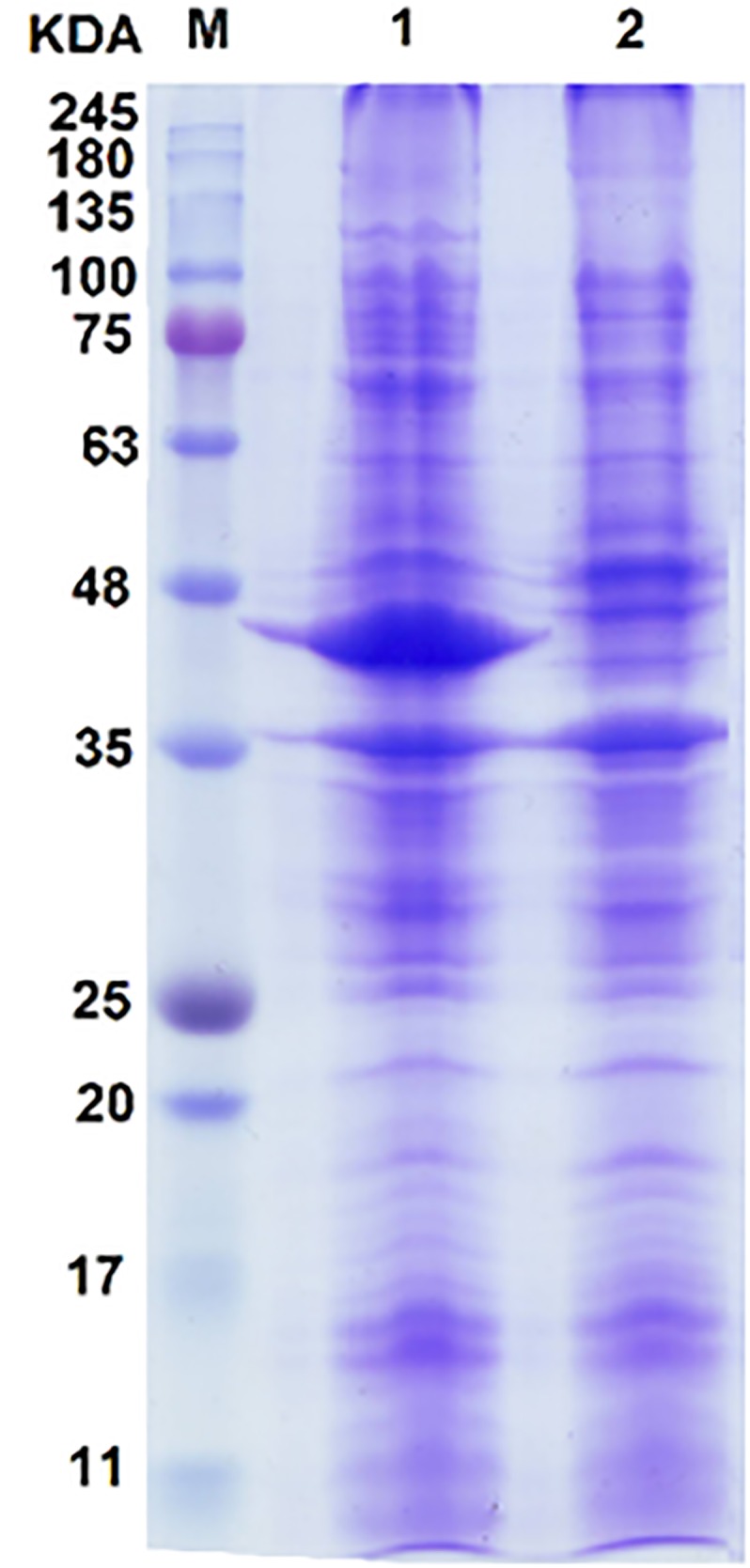
SDS–PAGE analysis of proteins resulting from the expression of the *dsz* gene cluster in *E. coli* BL21 host. Lane 1, induced recombinant cell; lane 2, negative control of *E. coli* BL21 carrying pCom8 without *dsz* genes; lane M, protein size marker. A diffused protein band with the size between 42 and 45 kDa is observed. This makes its accurate assignment rather difficult. Because of close protein sizes of DszA (45.1 kDa) and DszC (42.2 kDa), they are often observed with overlap. No protein band was observed on SDS–PAGE that can be referred to DszB with 38.9 kDa protein size (see text for possible explanations).

The SDS–PAGE result showed a prominent band referring to overproduced proteins (**Figure 2**). The estimated size of protein bandon gel was approximately 43 kDa. The size of deduced amino acid sequences for *dszA*, *dszB*, and *dszC* genes from FUM94 strain were calculated as 42.2, 38.9, and 45.1 kDa, respectively (Khosravinia et al., 2018). It should be noted that DszA protein of FUM94 was truncated and shorter than DszA of IGTS8 (with molecular weight 49.5 kDa). Whereas, DszB and DszC were identical in both strains (Gray et al., 1996). Since, the size of prominent band was between estimated sizes of DszA and DszC proteins from FUM94, and not well resolved to be assigned to a single protein, it could be related to DszA and DszC proteins, simultaneously. No band was observed on the gel close to DszB size. The possible explanation is that DszB protein was present in insoluble fraction indicating that it existed as inclusion bodies. It was reported that co-expression of *dszB* with chaperonin genes could increase the solubility of DszB provided that the cultivation temperature was reduced from 37 to 25°C (Nakayama et al., 2002). However, the greater part of the protein produced still remained in the insoluble fraction. There were two hypotheses for such observation, first, the folding intermediate of DszB was unstable at higher temperatures and second, the rapid accumulation of DszB increased the tendency to form inclusion bodies (Nakayama et al., 2002). It was notable that DszB protein was the most unstable in compared to DszA and DszC. DszB protein was easily inactivated in temperatures above 30°C, while DszC and DszA were stable at up to 40°C and 35°C, respectively (Gray et al., 1996; Ohshiro et al., 1997; Ohshiro et al., 1999).

### Desulfurization Activity of Recombinant Strain

Desulfurization activity of recombinant *E. coli* BL21 (DE3) strain was examined to determine the success of *dsz* genes insertion in pCom8 vector as well as the removal of sulfate repression. Two sets of biphasic reaction experiments were carried out using recombinant cells along with experiments using wild type *Rhodococcus* sp. FUM94 and negative control. In the first set, DBT was initially dissolved in ethanol as a co-solvent and then added to the two-phase system. Organic phase was pure *n*-tetradecane. In the second set of experiments, DBT was directly dissolved in *n*-tetradecane as organic phase. The first series of experiments were conducted since the gram negative recombinant *E. coli* reportedly uptakes sulfur source from aqueous phase rather than organic phase. Caro et al. (2007) indicated that in cases where DBT is dissolved in ethanol and then transferred into the biphasic system, part of DBT remains in aqueous phase that is beneficial to gram negative bacteria such as *Pseudomonas putida*. They indicated that these bacteria cannot uptake sulfur from hydrocarbon media. This is while gram positive *Rhodococcus* sp. FUM94 uptakes sulfur from organic phase very efficiently.

The reactions were conducted with 2.0 g L^-1^ resting cells dispersed in phosphate buffer (aqueous phase) and DBT concentrations of 25, 50, and 100 ppm in aqueous or organic phases. Ratio of aqueous phase to organic phase was 3:1.

**FIGURE 3:**
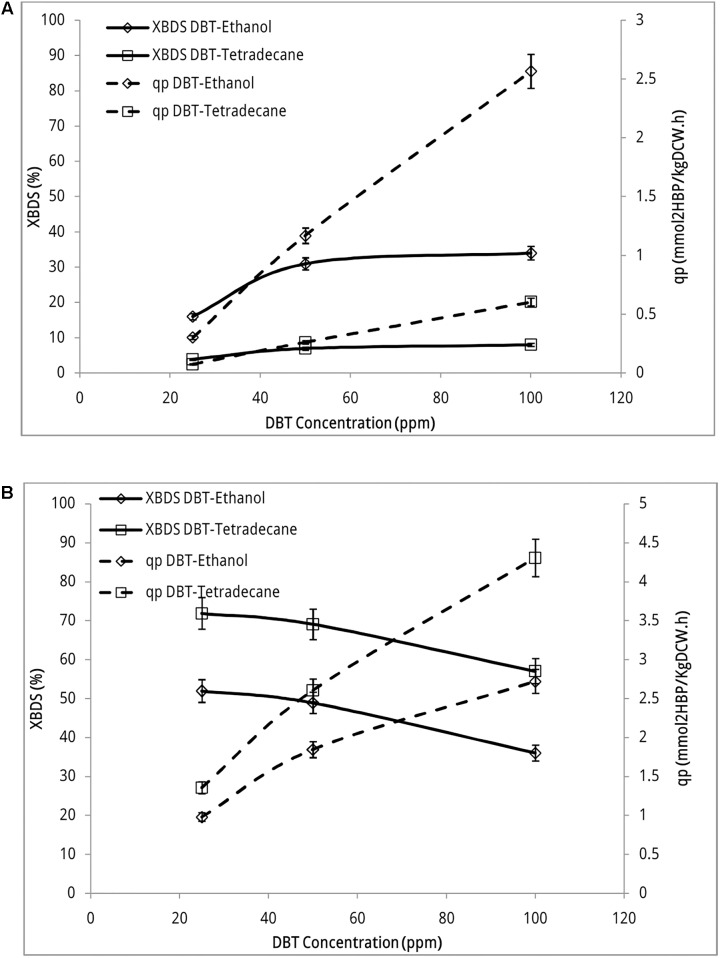
Desulfurization activity of recombinant *E. coli* BL21 strain **(A)** and *Rhodococcus* sp. FUM94 **(B)** at different DBT concentrations. “DBT-Ethanol” represents DBT dissolved in ethanol (aqueous phase) and “DBT-Tetradecane” represents DBT dissolved in *n*-tetradecane (organic phase). Aqueous to organic phase ratio: 3:1, reaction time: 12 h, biocatalyst concentration: 2 gl^-1^. 2-HBP specific production rate (q_p_) defined as the quantity of 2-HBP produced per hour per kilogram of dry cell weight, [mmol 2-HBP (kg DCW)^-1^ h^-1^] and BDS yield (X_BDS_) defined as the ratio of the 2-HBP produced to the initial substrate concentration.

**Figure 3A** indicates desulfurizing activity of recombinant strain in both sets of biphasic systems. This activity occurred while biocatalyst was grown in presence of sulfate (1.5 mM MgSO_4_ in BSM growth medium). Both BDS yield and 2-HBP production rate increased with the increase of DBT concentrations in the reaction. BDS yield increased from 16.0% ± 0.90 to 31.0% ± 1.7 when DBT concentration (dissolved in ethanol) increased from 25 ppm through 50 ppm. When substrate concentration increased from 50 ppm to 100 ppm slight change occurred and BDS yield increased to 34.0% ± 1.9. However, 2-HBP production rate increased from 0.302 ± 0.020 mmol 2-HBP (kg DCW)^-1^ h^-1^ at DBT concentration of 25 ppm to 2.57 ± 0.14 mmol 2-HBP (kg DCW)^-1^ h^-1^ at DBT concentration 100 ppm, which accounts for 8.5-fold product formation. These observations indicate that sulfur repression has been fully eliminated in the recombinant strain as it actively metabolized DBT even if it was grown in the presence of sulfate. The transcription of inserted PalkB promoter has no longer been repressed by sulfate and *dsz* genes are expressed.

In experiments that DBT was dissolved in *n*-tetradecane, BDS yield did not exceed more than 8.01% ± 0.45 in the whole range of DBT concentrations. Accordingly, 2-HBP production rate increased up to 0.605 ± 0.030 mmol 2-HBP (kg DCW)^-1^ h^-1^ at the highest concentration of DBT which is less than the lowest production rate of native strain. This is a clear indication that gram negative recombinant strain uptakes majority of its sulfur requirement from aqueous phase rather than organic phase. Mass transfer limitation significantly confines the uptake rate of sulfur from the interface.

**Figure 3B** indicates desulfurization activity of wild type *Rhodococcus* sp. FUM94 in sulfate-free BSM medium. No 2-HBP production was detected in FUM94 biphasic reaction when resting cell was prepared from a sulfate-containing BSM growth medium. As seen in the figure, both BDS yield and 2-HBP production rate are higher in cases where DBT is dissolved in *n*-tetradecane rather than ethanol. On average, the BDS yields in *n*-tetradecane-DBT reactions are 20% higher than those in ethanol-DBT reactions. However, in both sets of experiments BDS yield is descending with the increase of DBT concentration. The BDS yield of 71.9% ± 4.1 at DBT concentration of 25 ppm decreased to 57.0% ± 3.2 at DBT concentration of 100 ppm when DBT was dissolved in *n*-tetradecane. In case of ethanol solvent, BDS yield decreased from 52.0% ± 2.9 to 36.0% ± 2.0 with increasing DBT concentration. 2-HBP production rate increased from 1.36 ± 0.08 mmol 2-HBP (kg DCW)^-1^ h^-1^ to 4.31 ± 0.24 mmol 2-HBP (kg DCW)^-1^ h^-1^ when concentration of DBT dissolved in *n*-tetradecane increased from 25 to 100 ppm. When DBT is dissolved in ethanol, 2-HBP production rate increased from 0.980 ± 0.060 mmol 2-HBP (kg DCW)^-1^ h^-1^ to 2.72 ± 0.15 mmol 2-HBP (kg DCW)^-1^ h^-1^ accounting for nearly threefold more production of 2-HBP when DBT concentration increased from 25 ppm to 100 ppm in the reaction.

Simultaneously, negative control demonstrated no desulfurizing activity in all experiments because of the lack of *dsz* genes.

Nevertheless, comparing the results for two strains indicated that even though desulfurizing activity of recombinant strain was generally lower than that of wild type, the trend of desulfurization activity was ascending in recombinant with increasing DBT concentrations. BDS yield of native FUM94 decreased from 71.9% ± 4.1 to 57.0% ± 3.2 when DBT concentration (in *n*-tetradecane) changed from 25 to 100 ppm, respectively. At the same conditions, BDS yield of recombinant strain increased from 16.0% ± 0.9 to 34.0% ± 1.9 (DBT dissolved in ethanol). This observation indicates that the tolerance of the modified strain to high DBT concentrations is more than that of wild type as previously reported in the literature (Martinez et al., 2017). This is also a great advantage of the new construct as in real hydrocarbon media at large-scale, concentrations of organosulfur compounds such as DBT are much higher than those examined in this study.

The lower desulfurizing activity of recombinant may be attributed to nature of the host and is not related to the desulfurizing potential of the new construct, indeed. The *E. coli* is a gram negative bacterium that uptakes sulfur source (DBT) from aqueous phase. DBT solubility in water is extremely limited. Ethanol as a co-solvent mediates the solution of DBT in aqueous phase, but; the concentration of ethanol in the medium should not exceed 1% vol because of toxic effects. Once the DBT dissolved in ethanol enters two-phase reaction, part of DBT migrates to organic phase because of higher solubility in hydrocarbons and bioavailability of sulfur source to *E. coli* becomes more limited. Furthermore, mass transfer limitation prevents *E. coli* cells from assimilating sulfur from organic phase through the interface so that the maximum BDS yield of BL21 in DBT-Tetradecane reactions is 8.01% ± 0.45 and 2-HBP production rate is 0.605 ± 0.030 mmol 2-HBP (kg DCW)^-1^ h^-1^. Thus, desulfurizing activity of BL21 is confined to the remaining DBT concentration in aqueous phase.

Sensitivity of *E. coli* BL21 to mass transfer limitation is much more serious than that of FUM94. The results in **Figure 3A** indicate that BDS yield of BL21 in DBT-Tetradecane reactions (3.98% ± 0.22–8.01% ± 0.45) is, on average, four times lower than that of DBT-ethanol reactions (16.0% ± 0.9–34.0% ± 1.9). 2-HBP production rates also confirm this behavior so that q_p_ in DBT-Tetradecane reactions (0.0752 ± 0.0043 mmol 2-HBP (kg DCW)^-1^ h^-1^ to 0.605 ± 0.030) is four times lower than q_p_ in reactions where DBT is dissolved in ethanol (0.302 ± 0.020 mmol 2-HBP (kg DCW)^-1^ h^-1^ to 2.57 ± 0.14). This is while in FUM94 reactions, change from DBT-Tetradecane to DBT-Ethanol reduced BDS yields values 20%, and q_p_ values 30%, on average, across DBT concentrations.

Although *E. coli* is a well-known host in most of genetic manipulations and in this work desulfurizing enzymes were successfully expressed, it is not an ideal host for the case of desulfurization in an organic or hydrocarbon environment in terms of desulfurization level. Nevertheless, fast growth rate of *E. coli* compared to other strains, low-cost medium and nutrient, well-known genetic trait and extensive research work performed on this strain has made *E. coli* an appealing tool for genetic engineering studies including *dsz* genes insertion. Martinez et al. (2017) discussed that several gram negative bacteria have been used in BDS because of some metabolic and genetic properties such as metabolic versatility and easy genetic manipulation; but, they did not address mass transfer limitation in gram negative systems that preferably uptake sulfur source from aqueous phase rather than organic phase. Practically, mass transfer limitation is the main cause of low BDS yield since majority of DBT is dissolved in organic phase and only insignificant amount of DBT remains in aqueous phase.

Mass transfer limitation comes from the low cell surface hydrophobicity (CSH) properties of gram negative *E. coli* BL21. Goldberg et al. (1990) reported that adhesion of *E. coli* cells to *n*-hexadecane is near zero. This property prevents *E. coli* cells from adhering to the interface of two phases and uptake sulfur source from organic phase. This is while the bacterial adhesion to hydrocarbons for gram positive *Rhodococcus* strains is higher than 78% (Rosenberg, 1984). This means that *Rhodococci* possess such properties that are favorable for attachment to hydrocarbon droplets in the interface and assimilate sulfur source. As a result, to improve the desulfurization potential of a recombinant biocatalyst, not only the sulfur repression of promoter transcription need to be removed, but also transfer of the modified gene cluster to a host with appropriate CSH need to be considered. High CSH facilitates the adhesion of cells to hydrocarbons in the organic phase and reduces mass transfer limitation to high extent.

Although mass transfer limitation reduces the transformation rate of organosulfur compounds such as DBT, it has the advantage of reusability of biocatalyst in biphasic systems at large-scale applications. In a continuous mode of operation, biocatalyst can be grown in a bioreactor on a growth medium supplemented with inorganic sulfur due to the removal of sulfur repression. Then the resting cell can be supplied to a reactor for two-phase desulfurization reaction. Upon the completion of reaction, biocatalyst can be easily separated and recycled back to the bioreactor to be enriched and reused. Reusability of biocatalyst in biphasic systems reduces the BDS cost and makes this process more attractive from the economic point of view.

Overall, the constraint of sulfate repression of native *dsz* promoter transcription was resolved by inserting PalkB promoter in *dsz* operon. Expression of Dsz enzymes in *E coli* BL21 demonstrated the success of cloning; and desulfurization of recombinant strain proved the removal of transcription repression. This removal not only enabled the recombinant strain to metabolize organosulfur DBT in both aqueous and organic phase, but also this capability increased with the increase of DBT concentration so that BDS yield increased twice and 2-HBP production rate increased more than eightfold with the increase of DBT concentration. Although the desulfurization capacity of wild type is still higher than that of recombinant strain in both phases, the ascending trend of desulfurization activity by recombinant strain compared to descending trend of that of wild type in presence of organic solvent is promising and is an indication of recombinant’s potential for desulfurization at high DBT concentrations. The lower activity of recombinant cell, as described earlier, is attributed to host physiological properties and adverse effect of mass transfer. Thus, finding an appropriate host that is compatible with hydrocarbon environment is still need to be further investigated.

Furthermore, repression of *dsz* promoter transcription in presence of inorganic sulfur in the culture medium has long been known as one of the reasons for low biocatalyst activity in desulfurizing bacteria (Li et al., 1996; Tanaka et al., 2002; Kirkwood et al., 2007). This problem has already been addressed in several publications using different molecular biology techniques including native promoter replacement and there are several examples of hosts having increased desulfurization activities while they contain replaced *dsz* promoter (Noda et al., 2003; Alves et al., 2007). But, it should be noted that in some of these examples (Shavandi et al., 2009) enhancement of activity was observed due to the self cloning rather than heterologous recombination. Also, in all previous studies the replacing promoter was not responsive to alkane environment (Piddington et al., 1995; Matsui et al., 2002) that is very similar to real diesel environment. In this study, for the first time, an alkane responsive vector (pCom8) harboring PalkB promoter was used for recombination of *dsz* genes. This vector has already been used for other purposes (van Beilen et al., 2003; Koch et al., 2009; Cornelissen et al., 2011; Liu et al., 2011), However, this is the first time that this vector was used for *dsz* insertion aimed at desulfurization. Moreover, since this vector with an alkane tolerant promoter was longer than normal promoters (nearly 11 kbps), cloning of *dsz* genes in this vector and then expressing of the new construct in *E. coli* BL21 as the host was quite challenging. Thus, successful cloning and expression of such suitable recombinant vector in *E. coli* is of interest in research community.

## Conclusion

Transcription repression of native *dsz* promoter in desulfurizing bacteria such as *Rhodococcus* sp. FUM94 was resolved by cloning *dsz* genes of wild type strain in an alkane responsive vector harboring PalkB promoter and expressing in gram negative *E. coli* BL21 host. The recombinant strain not only was no longer repressed by inorganic sulfate, but also demonstrated higher tolerance in DBT environment than wild type, so that desulfurization activity of recombinant strain increased with increasing DBT concentration, while in case of wild type otherwise behavior observed. The recombinant metabolized DBT in aqueous phase better than organic phase and the wild type demonstrated higher DBT degradation in organic phase.

## Author Contributions

SK conducted the experiment. MM designed and supervised the study. RG contributed in the experimental setup. HD supervised parts of the laboratory work and contributed in the interpretation of results. BR isolated the strain FUM94.

## Conflict of Interest Statement

The authors declare that the research was conducted in the absence of any commercial or financial relationships that could be construed as a potential conflict of interest.
